# Controllable Resistive Switching in ReS_2_/WS_2_ Heterostructure for Nonvolatile Memory and Synaptic Simulation

**DOI:** 10.1002/advs.202302813

**Published:** 2023-08-02

**Authors:** Feihong Huang, Congming Ke, Jinan Li, Li Chen, Jun Yin, Xu Li, Zhiming Wu, Chunmiao Zhang, Feiya Xu, Yaping Wu, Junyong Kang

**Affiliations:** ^1^ Department of Physics Engineering Research Centre for Micro‐Nano Optoelectronic Materials and Devices at Education Ministry Fujian Provincial Key Laboratory of Semiconductor Materials and Applications Xiamen University Xiamen 361005 P. R. China; ^2^ Ningbo Institute of Materials Technology and Engineering Chinese Academy of Sciences Ningbo 315211 P. R. China; ^3^ Pen‐Tung Sah Institute of Micro‐Nano Science and Technology Xiamen University Xiamen 361005 P. R. China

**Keywords:** 2D memristor, biological synaptic functions, gate and optical controllability, van der Waals heterostructure

## Abstract

Memristors with nonvolatile storage performance and simulated synaptic functions are regarded as one of the critical devices to overcome the bottleneck in traditional von Neumann computer architecture. 2D van der Waals heterostructures have paved a new way for the development of advanced memristors by integrating the intriguing features of different materials and offering additional controllability over their optoelectronic properties. Herein, planar memristors with both electrical and optical tunability based on ReS_2_/WS_2_ van der Waals heterostructure are demonstrated. The devices show unique unipolar nonvolatile behavior with high *R*_off_/*R*_on_ ratio of up to 10^6^, desirable endurance, and retention, which are superior to pure ReS_2_ and WS_2_ devices. When decreasing the channel length, the set voltage can be notably reduced while the high *R*_off_/*R*_on_ ratios are retained. By introducing electrostatic doping through the gate control, the set voltage can be tailored in a wide range from 4.50 to 0.40 V. Furthermore, biological synaptic functions and plasticity, including spike rate‐dependent plasticity and paired‐pulse facilitation, are successfully realized. By employing optical illumination, resistive switching can also be modulated, which is dependent on the illumination energy and power. A mechanism related to the interlayer charge transfer controlled by optical excitation is revealed.

## Graphical abstract

Scalable 2D planar memristors based on monolayer ReS_2_/WS_2_ van der Waals heterostructure are fabricated. The devices show superior nonvolatile memristive properties and simulated synaptic functions. Both the electrical and optical modulations on the resistance states and programming voltage are realized. The resistance switching behavior and electrical and optical controllability are attributed to the unique optoelectronic properties and interlayer interaction of the heterostructure.

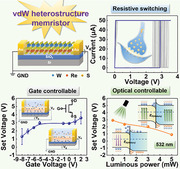

## 1 Introduction

Recent years have witnessed a spurt of progress in computing and communications technology. As traditional silicon‐based microelectronic devices have struggled to scale down further according to Moore's Law, advanced storage technologies require new materials and device designs.^[^ ^1^, ^2^ ^]^ Memristors can maintain the internal resistance state created by the applied voltage and current,^[^ ^3^, ^4^ ^]^ which makes them viable to conduct memory computing without any program and emerging building blocks of artificial neural networks. Compared with the early neuromorphic computing based on complementary metal‐oxide‐semiconductor transistors, using memristors to construct synapses and neurons can greatly reduce computational energy consumption.^[^ ^5^ ^]^

The preliminary study of memristors mainly involved traditional 3D bulk materials and thin films,^[^ ^6^ ^]^ which are challenging to reduce the size to meet the stringent requirements of future big data and artificial intelligence for high‐density integration and low power consumption. 2D semiconductors are considered to be a focal point for future research due to their ultra‐thin thickness, high flexibility, and intriguing optoelectronic properties.^[^ ^7^, ^8^ ^]^ Rhenium disulfide (ReS_2_) is a promising 2D material with unique physical and chemical properties. The soft Re─S covalent bonds are more likely to produce sulfur (S) vacancies, providing an intensive possibility for the development of 2D memristors. The S vacancies have an even lower forming energy and more evident movement under the applied bias,^[^ ^9^, ^10^, ^11^ ^]^ which are beneficial to improve the switching performance of memristors.^[^ ^10^ ^]^ Li et al. demonstrated a double‐ended lateral memristor based on a few‐layer ReS_2_, realizing an amnestic block function with a switching ratio of 10^2^,^[^ ^12^ ^]^ and further implementing a simulation of synaptic plasticity.

Nevertheless, the retention and durability of memristors based on a single 2D material may be relatively lower than that of traditional 3D devices. Heterostructures can integrate the properties of different materials, which have become an alternative to enhance the performance of memristors.^[^ ^13^, ^14^ ^]^ In order to take advantage of the excellent properties of 2D materials and also consider the compatibility with conventional microelectronic technology, attention has been targeted to 2D/2D heterostructures with steep potential gradients at the atomic level. The stacked layers in the heterostructures are no longer restricted by lattice matching due to their van der Waals interface. Zhang et al. designed a 2D/2D memristor with a few‐layer MoS_2_/WS_2_ heterostructure, implementing a resistive switching mechanism based on the band structure modulation with an *R*_off_/*R*_on_ ratio of 10^4^.^[^ ^15^ ^]^ Its higher stability, larger memory window, and superiority in high‐density integration further confirm the great potential of 2D van der Waals heterostructures for memristor applications.

In addition, most of the existing memristors are solely based on electrical modulation. It does not completely match the practical human stimuli, including vision, hearing, touch, smell, and so on. Light as an extra stimulus may also supply a way to fill the gap between the visual system and brain function.^[^ ^16^ ^]^ Beyond pure electronic devices, further introducing the optical signals as information carriers during the device's operation has advantages in terms of high bandwidth, low crosstalk, and high connectivity. Optical programmability also facilitates the applications in the field of neuromorphic vision.^[^ ^17^ ^]^ Therefore, developing a memristor with both electrical and optical controllability is significantly crucial. In this regard, van der Waals heterostructures possess the advantage of modulating the optical absorption and exciton recombination through the design of their band alignments.^[^ ^18^, ^19^ ^]^

In this work, 2D planar memristor are fabricated based on ReS_2_/WS_2_ heterostructure through an alignment transfer of chemical vapor deposition (CVD) grown monolayers. The lattice configuration, stacking structure, and band alignment are determined by Raman scattering, transmission electron microscopy (TEM), time‐resolved photoluminescence (TRPL), and Kelvin probe force microscopy (KPFM). The memristive properties, such as resistance states, switching ratio, cycle number, and retention time, are investigated at room temperature and compared with that of pure ReS_2_ and WS_2_ devices. The dependences of resistive switching properties and *R*_off_/*R*_on_ ratios on the channel length of the heterostructure memristor are studied. The synaptic functions of short‐term plasticity (STP), spike rate‐dependent plasticity (SRDP), and double‐pulse (PPF) dissimilation are imitated. Moreover, the unique gate and optical control over the resistive switching are achieved, and the related mechanisms are further revealed.

## 2 Results and Discussion

**Figure 1:**
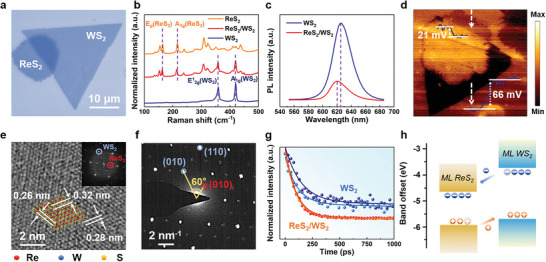
Morphological, spectroscopic, and structural characterizations. a) Optical image of a transferred ReS_2_/WS_2_ heterostructure on SiO_2_/Si substrate. b) Raman spectra of the ReS_2_, WS_2_, and ReS_2_/WS_2_ regions. c) PL spectra of the WS_2_ and ReS_2_/WS_2_ regions. d) KPFM image of the ReS_2_/WS_2_ heterostructure, with the potential profile taken along the white dashed arrows. e) High‐resolution transmission electron microscopy (HRTEM) image of the ReS_2_/WS_2_ heterostructure, with the inset showing the fast Fourier transform (FFT) image. f) Selected area electron diffraction (SAED) patterns of the ReS_2_/WS_2_ heterostructure. g) Decay curves of the TRPL measurements for pristine WS_2_ and ReS_2_/WS_2_ heterostructure (each case is repeated twice to ensure accuracy). h) Proposed schematic diagram of the band alignment and the charge transfer in the heterostructure.

For the development of ReS_2_/WS_2_ memristors, the properties of ReS_2_/WS_2_ heterostructure are studied first. The heterostructure is prepared through an alignment transfer of CVD‐grown ReS_2_ and WS_2_ monolayers (as detailed in the Experimental Section). The optical microscopy in **Figure** **1**a shows clear edges all around the sample, and the heterostructure is vertically stacked with triangular WS_2_ on top of hexagonal ReS_2_. The crystal and electronic structures of the ReS_2_/WS_2_ region are characterized through the Raman and photoluminescence (PL) measurements. As shown in Figure 1b, the Raman spectrum of the heterostructure region coincides with the combination of characteristic peaks from the two monolayers. The peaks located at 164 and 216 cm^−1^, respectively, correspond to the in‐plane (E^1^_2g_) and out‐of‐plane (A_1g_) vibrational modes from ReS_2_,^[^ ^11^ ^]^ respectively, the two peaks at 359 and 420 cm^−1^ are identified to be from monolayer WS_2_.^[^ ^20^, ^21^, ^22^ ^]^ Besides, a series of other modes arise due to the symmetry splitting in the distorted 1T structure of ReS_2_.^[^ ^23^ ^]^ Different from the superposition of the Raman peaks, the PL spectrum for the heterostructure demonstrates an obvious intensity decay and a 5 nm blue shift with respect to the monolayer WS_2_ (625 nm), as shown in Figure 1c, which suggests an interlayer charge transfer and stress interaction.^[^ ^24^, ^25^ ^]^ The intensity mappings of Raman and PL peaks further reveal the good uniformity of the structures (Figure S1a–c in the Supporting Information). Accordingly, we infer that a type II heterostructure may be formed between ReS_2_ and WS_2_.^[^ ^26^ ^]^ The atomic force microscope (AFM) image (Figure S1d in the Supporting Information) also indicates the existence of interlayer interaction.^[^ ^27^ ^]^

Interlayer interaction related to the band alignment of WS_2_ and ReS_2_ monolayers is closely associated with the electronic properties of the heterostructure. To gain an insight into their band alignment, KPFM is employed to detect the work function (*φ*_s_) of a 60° stacked ReS_2_/WS_2_ heterostructure on an Au‐plated SiO_2_/Si substrate. As shown in Figure 1d and Figure S2a,b in the Supporting Information, a clear variation of *φ*_s_ between ReS_2_, WS_2_, and the heterostructure areas is observed, in good agreement with the optical contrast. By measuring the potential change in ReS_2_/Au and WS_2_/Au steps, the *φ*_s_ difference is determined to be about −21 mV between ReS_2_ and Au, and about −66 mV between WS_2_ and Au, which indicate a higher Fermi level of WS_2_ than that of ReS_2_ (details in Figure S2c,d in the Supporting Information). HRTEM characterization (detailed in the Experimental Section) is further performed to investigate the lattice and stacking structures of the ReS_2_/WS_2_ heterostructure. As measured in Figure 1e, the space between two Re atoms in the Re4‐chain (0.26 nm) is smaller than that of adjacent two Re atoms (0.32 nm), consistent with a 1T'‐ReS_2_ structure, while the distance between neighboring W atoms (0.28 nm) corresponds to 2H‐WS_2_.^[^ ^28^ ^]^ The FFT image (insert in Figure 1e) and SAED patterns (Figure 1f) contain two sets of sixfold symmetrical lattices, corresponding to the lattice spacings of 5.5 and 2.8 Å, respectively. The lattice structures are identified to the (010) planes of monolayer WS_2_ and ReS_2_, respectively, confirming a 60° vertical stacking of the heterostructure.

Photoinduced interfacial charge behavior is studied through the TRPL measurements (Figure 1g). The excited WS_2_ in ReS_2_/WS_2_ heterostructure processes three physical processes, the nonradiative surface state recombination, the radiative recombination, and the interlayer charge transfer. Therefore, its TRPL curve can be fitted by the multiexponential function

(1) $$D\;\left(t\right)=A_{1}{\;e}^{-\frac{t}{\tau_{1}}}+A_{2}e^{-\frac{t}{\tau_{2}}}+A_{3}e^{-\frac{t}{\tau_{3}}}$$

where *D*(*t*) is the exciton concentration, *τ_i_* (*i* = 1, 2, 3) are the time constants (the shortest and longest ones correspond to nonradiative recombination and radiative recombination, respectively, and the middle one corresponds to charge transfer), and *A_i_* (*i* = 1, 2, 3) correspondingly symbolizes the changes in exciton density due to the above three physical processes, respectively.^[^ ^29^ ^]^ While in pristine monolayer WS_2_, the interlayer charge transfer does not exist, so its TRPL curve is fitted by a biexponential function. As the results show in Table S1 in the Supporting Information, the lifetime of ReS_2_/WS_2_ heterostructure (about 134 ps) is found to be significantly shorter than that of monolayer WS_2_ (about 700 ps). This conforms to the property of type II band alignment, which is further confirmed by the first‐principles calculations in Figure S3 in the Supporting Information. Accordingly, the band structure is schematically shown in Figure 1h, where the conduction band minimum and valence band maximum locate in WS_2_ and ReS_2_, respectively. The facilitated interlayer charge transfer strongly predicts a possibility of electrical modulation for the heterostructure.

**Figure 2:**
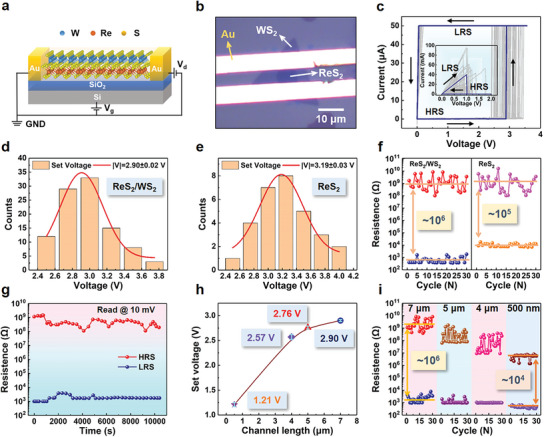
Resistive switching performance of the ReS_2_/WS_2_‐based planar memristor. a,b) Schematic diagram and optical micrograph, respectively. c) *I*–*V* curves of the ReS_2_/WS_2_‐based planar memristor showing unipolar resistive switching behavior; the inset shows the resetting *I*–*V* curves. d,e) Statistical distributions of the set voltages for ReS_2_/WS_2_‐based and ReS_2_‐based planar memristors, respectively. f) Comparison of both the LRS and HRS between ReS_2_/WS_2_‐based and ReS_2_‐based planar memristors. g) Statistical analysis of the retention times recorded at the LRS and HRS for the ReS_2_/WS_2_‐based planar memristor. h) Channel length‐dependent *V*_set_ of ReS_2_/WS_2_‐based planar memristor. i) Comparison of the LRS and HRS of ReS_2_/WS_2_‐based planar memristors with channel lengths of 7, 5, 4 µm, and 500 nm.

Based on the understanding of the electronic properties, the ReS_2_/WS_2_‐based planar memristor is constructed on a SiO_2_/Si substrate using Au as the source and drain electrodes. Each electrode is in contact with both the ReS_2_ and WS_2_ monolayers simultaneously, as shown in the schematic diagram and the optical image in **Figure** **2**a,b. The *I–V* characteristics of the device exhibit a typical resistive switching behavior, as shown in the blue curve in Figure 2c. During the measurements, a compliance current is set at 50 µA (which can be controlled by connecting series resistors or modifying device structures in practical applications^[^ ^30^ ^]^), followed by a voltage sweeping from 0 to 4 V. At an applied voltage of about 3 V, the current increases abruptly, completing the “SET” process from high resistance state (HRS) to low resistance state (LRS); after a voltage sweeping from 4 V back to 0 V, the device remains LRS. By removing the compliance current setting and performing a voltage sweep from 0 to 2 V, the device shifts from LRS to HRS at an increasing current, corresponding to the “RESET” process, and the device maintains HRS at the reversed sweep from 2 to 0 V. Similar performance of the device is found under the negative voltages, as shown in Figure S4 in the Supporting Information. This electrical property confirms a unipolar memristive switching behavior. It may be attributed to the greater Joule heat effect than that of the electric field in breaking the conductive channel because the CVD‐grown materials can contain more S vacancies than the mechanical exfoliated samples and thus generate larger Joule heat as the current increases. Different from the previously reported ReS_2_ memristor that mostly exhibited bipolar resistive switching behavior,^[^ ^12^, ^31^, ^32^ ^]^ the ReS_2_/WS_2_ unipolar memristor predicts a higher switching ratio, higher integration density, and more simplified control circuit. Notable reliability is demonstrated during the repeated switching for 100 cycles (the gray cycle curves in Figure 2c). The set voltage (*V*_set_) is extracted and depicted in the histogram in Figure 2d. A Gaussian fit suggests that the *V*_set_ generally distributes around 2.90 V. The high uniformity and stability of the device performance are confirmed by the device‐to‐device statistic results, as shown in Figure S5 in the Supporting Information. The nonvolatile electrical characteristic of the devices is further verified by the comparison of *I–V* curves measured after storage in air for 1, 47, 69, 195, 281, and 342 days (Figure S6a,b, Supporting Information).

For comparison, the electrical properties of the devices based on pristine monolayer ReS_2_ and WS_2_ are also studied and shown in Figure S7 in the Supporting Information, respectively. The ReS_2_‐based device exhibits a typical unipolar memristive property, where the resistance jumps from HRS to LRS at a *V*_set_ of about 3.19 V, as depicted in the histogram in Figure 2e. While for the WS_2_‐based device, the HRS is maintained without significant change even under a voltage exceeding 7 V. Obviously, the ReS_2_ plays a key role in the resistive switching for the heterostructure since the ReS_2_ and WS_2_ layers are in parallel connecting to the Au electrons. The current for ReS_2_ before switching is about an order of magnitude larger than WS_2_, and after switching is much higher further. Therefore, it can be inferred for ReS_2_/WS_2_ heterostructure that most of the current passes through the ReS_2_ region. This suggests that the affect from the parallel WS_2_ region is very small that could not influence the main results. By comparison, the ReS_2_/WS_2_‐based planar memristor has a decreased and more stable *V*_set_ value than monolayer ReS_2_.

Except for the *V*_set_, another crucial factor determining the overall performance of a memristor is the memory window that is reflected as *R*_off_/*R*_on_ ratio between HRS and LRS. As shown in Figure 2f, the ReS_2_/WS_2_‐based memristor exhibits a notable *R*_off_/*R*_on_ ratio higher than 10^6^, which is superior to most reported 2D memristors.^[^ ^12^, ^27^, ^33^, ^34^, ^35^ ^]^ This value is more than an order of magnitude larger than pure ReS_2_ (10^5^), indicating a larger memory window when forming the heterostructure. Subsequently, a switching cycling test of the heterostructure memristor is conducted over 200 times (Figure S6c, Supporting Information), which shows a reliable resistive switching performance with a clear memory window. Desirable stability of the memristor is also evidenced by the retention time of over 10^4^ s for each HRS and LRS, as seen in Figure 2g.

In order to study the dimension effect, the resistive switching properties and *R*_off_/*R*_on_ ratios of ReS_2_/WS_2_‐based memristors with different channel lengths are measured and compared in Figure S8 in the Supporting Information and Figure 2h,i. All the devices exhibit typical unipolar memristor characteristics (Figure S8b,e,h, Supporting Information) with high reliability during the repeated switching cycles. The *V*_set_ generally distributes around 2.90, 2.76, 2.57, and 1.21 V, exhibiting a decreasing trend with reducing channel length from 7 to 5, 4 µm, and 500 nm (Figure 2g and Figure S8c,f,i, Supporting Information). Moreover, as shown in Figure 2i and Table S2 in the Supporting Information, the LRS decreases slightly from ≈2.5 × 10^3^ to 5 × 10^2^ Ω, while the HRS decreases obviously from ≈2.5 × 10^9^ to 6 × 10^6^ Ω as the channel length decreases. Before the switching, the conductivity mainly originates from both the defects and electrons within the materials. The electrons are more easily routed with a smaller channel length (similar to the short‐channel effect). Therefore, the HRS is seriously decreased with decreasing the channel length. After the switching, the conductive channels are mostly formed by the S vacancies, the conductivity for different channel lengths becomes comparable, and the variation of LRS is much smaller. Therefore, the *R*_off_/*R*_on_ ratio reduces for the smaller channel lengths, and the decreased HRS is primarily responsible for it. Even if the channel length is reduced to 500 nm, the *R*_off_/*R*_on_ ratio can still reach ≈10^4^, exhibiting a magnitude improvement for the ReS_2_/WS_2_‐based memristors compared with the ReS_2_ ones.^[^ ^12^ ^]^

**Figure 3:**
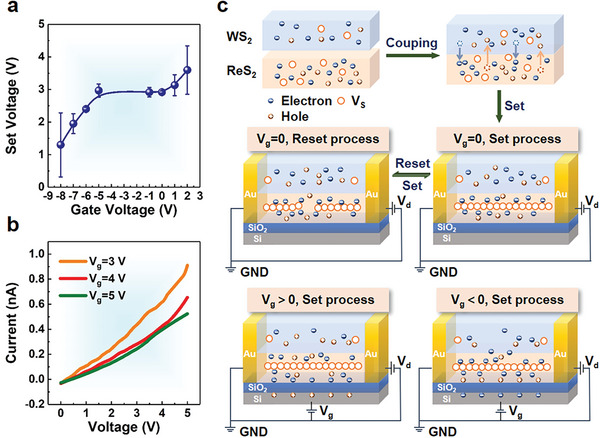
a) Gate tunable *V*_set_ of the ReS_2_/WS_2_‐based planar memristor. b) *I*–*V* curves of the memristor at the gate voltages of 3, 4, and 5 V. c) Schematic diagrams of the resistive switching mechanism for the ReS_2_/WS_2_‐based planar memristor.

Furthermore, the electrical modulation capability is examined by the three‐terminal field effect transistor configuration. The *I–V* curves under different gate voltages (*V*_g_) are measured (Figure S9, Supporting Information), and the *V*_g_‐dependent *V*_set_ is summarized in **Figure** **3**a. As is shown, opposite variation trends for the *V*_set_ are found under different *V*_g_ directions. For positive gating (*V*_g_ > 0), the *V*_set_ increases from 2.90 to 4.50 V when increasing the *V*_g_ from 0 to 2 V. As the positive *V*_g_ increases to 3, 4, and 5 V, the device is always in an HRS within the voltage sweep range of 0–5 V, as shown in Figure 3b, i.e., the device is blocked from turning on. To be more specific, a negative gating (*V*_g_ < 0) significantly reduces the *V*_set_ of the device, which is decreased from 2.90 to 0.40 V when changing the *V*_g_ from −5 to −8 V. While in the *V*_g_ range of 0 to −5 V, the *V*_set_ is relatively stable. Therefore, the ReS_2_/WS_2_‐based memristors not only possesses a high *R*_off_/*R*_on_ ratio, good endurance, and desirable retention but also demonstrates significant gate controllability. This holds great potential for their applications in logic devices or other relative fields.^[^ ^36^ ^]^

The mechanism of resistive switching and gate modulation for the ReS_2_/WS_2_‐based planar memristor is described in Figure 3c. Monolayer ReS_2_ and WS_2_ grown by the CVD method have been demonstrated to possess n‐type conductivity with intrinsic S vacancies and additional electrons.^[^ ^37^, ^38^ ^]^ When forming the heterostructure, the electrons will transfer from WS_2_ to ReS_2_ owing to the type II band alignment (the top panel in Figure 3c). By applying a forward bias and a compliance current, the ReS_2_ layer transports electrons to the conducting channel to form a current. When the bias voltage reaches a threshold, the conducting channel meets the condition for rapid migration of electrons between electrodes, and the device jumps from HRS to LRS (“SET” process, the middle panel in Figure 3c). Hence, the formation of conducting filaments by S vacancies is the primary conduction mechanism in the memristor.^[^ ^12^ ^]^ The as‐grown ReS_2_ generally possesses a lower stoichiometric ratio than that of WS_2_ and thus has a greater possibility to generate S vacancies.^[^ ^12^, ^39^ ^]^ As such, the resistive switching is dominant by the ReS_2_ layer during the device operation, while the WS_2_ layer maintains HRS. The intense accumulation of electrons near the beginning of the conducting channel causes a noticeable reduction of interface electrons in the ReS_2_ layer. Therefore, more electrons will transfer from WS_2_ to ReS_2_, which explains the faster conductance change of the ReS_2_/WS_2_‐based device than that of ReS_2_. Simultaneously, owing to the addition of electrons, the resistance at LRS of the ReS_2_/WS_2_‐based device is an order of magnitude lower (Figure 2e), resulting in its higher *R*_off_/*R*_on_ ratio. When the voltage with the same polarity is scanned again without current compliance, the effect of Joule heating is greater than that of the voltage between electrodes. As a result, the conducting channel breaks, and the S vacancies gradually return to their original state.^[^ ^40^ ^]^ The device resistance jumps back to the HRS (“RESET” process).

When applying a positive *V*_g_ during the “SET” process, some electrons in the device are attracted to the interface of ReS_2_ and SiO_2_ substrate due to the electrostatic equilibrium effect (the bottom panel in Figure 3c). Fewer electrons could jump around the conducting channel made up of S vacancies, causing an increased *V*_set_ threshold required for the resistive switching (Figure 3a). As the *V*_g_ increases further, the consumption of electrons finally blocks out the device from turning on (Figure 3b). In contrast, by applying a negative *V*_g_, the electrostatic equilibrium effect causes the holes to accumulate at the interface near the SiO_2_ substrate. In a small gating range of 0 to −5 V, the consumed holes in ReS_2_ can be supplemented by those transferred from WS_2_, leading to a relatively stable *V*_set_. Further consumption of holes results in more net electrons for electrical conduction. The left electrons are active to the conducting channel formed by S vacancies, and the required *V*_set_ threshold thus gradually decreases (Figure 3a).

**Figure 4:**
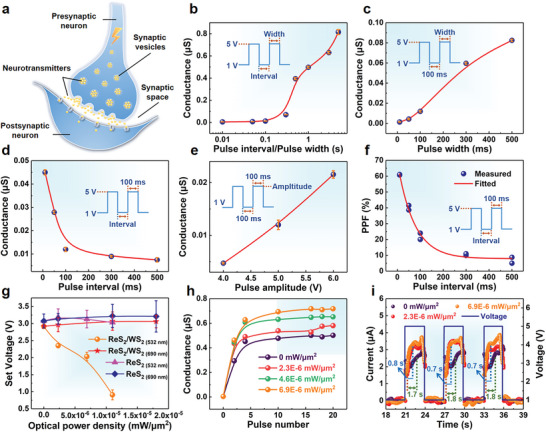
Synaptic functions of the ReS_2_/WS_2_‐based planar memristor. a) Schematic demonstration of presynaptic and postsynaptic neuron‐based synaptic functions. b) The effect of simultaneously varying pulse width and interval on device conductance. c–e) Conductance modulation with different pulse widths, intervals, and amplitudes. f) PPF ratio as a function of two sequential pulse intervals. g) Impact of different optical power densities on the *V*_set_ of ReS_2_/WS_2_‐based and ReS_2_‐based devices under 532 and 690 nm lasers, respectively. h) Relationship between conductance and optical power densities for ReS_2_/WS_2_‐based device at 1 s pulse width and interval. i) Modulation of current stability under different optical power densities for ReS_2_/WS_2_‐based device at 3 s pulse width and interval.

The superior electronic performance of the ReS_2_/WS_2_‐based planar memristor sheds light on exploring its potential applications. We then demonstrate that it can simulate partial neuron‐based biological synaptic functions. As shown in **Figure** **4**a, when a presynaptic neuron is stimulated and conducted to a synaptic vesicle, the synaptic vesicle fuses tightly with the presynaptic membrane and causes a rupture. The neurotransmitters within the synaptic vesicles are released into the synaptic space, diffuse to reach the postsynaptic membrane, and thereby induce excitatory or inhibitory modifications in the postsynaptic membrane. For the ReS_2_/WS_2_‐based planar memristor, the source extreme of the memristor can serve as the applying end of the presynaptic stimulus, the S vacancies are comparable to neurotransmitters in biological synapses, and a pulse voltage simulates the stimulus signal, which will result in a corresponding excitatory postsynaptic current. Therefore, the conductance of the memristor can be modulated by varying the pulse parameters, such as width, interval, and amplitude, to adjust the synaptic weights.

As shown in Figure S10 in the Supporting Information, at a 1 V reading voltage and a fixed 5 V amplitude, the device current tends to saturate with the continuous application of pulse when the pulse width and interval increase in equal proportion. This performance exhibits a typical memristor character.^[^ ^41^ ^]^ Accordingly, the device property is analyzed by acquiring the conductance at the saturated currents for different pulse amplitudes, widths, and intervals. As shown in Figure 4b, the conductance is increased when simultaneously increasing the pulse width and interval from 10 ms to 5 s, indicating a positive response to a continuously longer stimulation. Moreover, the maximum/minimum conductance ratio is found to be closely related to the pulse width and interval. When simultaneously increasing the pulse width and interval from 10 ms to 5 s, the maximum/minimum conductance ratio can reach more than 185 (the minimum value is 0.0044 µS at the pulse interval/width of 0.01). The conductance can also be modulated by individually adjusting the pulse width or interval. An enlarged pulse width increases the upward trend of the conductance, while an opposite trend is found when increasing the pulse interval, as shown in Figure 4c,d. Modulating the memristor characteristics by adjusting pulse width and interval is recognized as the biological synapse SRDP.^[^ ^42^ ^]^ The maximum/minimum conductance ratio can reach 9, 46, and 63 for the pulse width of 100, 300, and 500 ms, respectively (the minimum value is 0.0014 µS at the pulse width of 10 ms). In addition, the impact of voltage amplitude is shown in Figure 4e, where the device conductance is increased with the enhancing pulse voltage. These results can be explained by the modulation of conductive filaments constructed by S vacancies because the migration of S vacancies is driven by the electric field. For a larger pulse width or a smaller pulse interval, the S vacancies can obtain more sustained electric stimulation within a certain time, which will promote their migration, widening the conductive filaments and leading to increased conductance. Similarly, an increased pulse amplitude will provide a larger electric field, which can also drive more S vacancies to form the conductive filaments and increase the conductance.

PPF representing the STP is an important physiological phenomenon. It is manifested as the temporal sum of biological synaptic inputs and can be estimated from the change of synaptic weights as responding to the stimuli of two consecutive pulsed voltages.^[^ ^43^, ^44^ ^]^ Figure 4f shows the PPF behavior of the ReS_2_/WS_2_‐based memristor, and the inset illustrates the applied pulse. The PPF can be quantitatively expressed as^[^ ^45^ ^]^

(2) $$\mathrm{PPF}=\frac{\left(B_{2}-B_{1}\right)}{B_{1}}\times 100\%$$

where *B*_1_ and *B*_2_ are the conductance corresponding to the first and second pulses, respectively. The fitting relationship between PPF data and pulse is as follows (Figure 4f)^[^ ^46^ ^]^

(3) $$y=C_{1}{\;e}^{-\frac{t}{\alpha_{1}}}+C_{2}e^{-}\frac{t}{\alpha_{2}}$$

which gives *α*_1_ = 0.06 s and *α*_2_ = 0.96 s, corresponding to the fast and slow decay terms, respectively. The enhancement of conductance under successive pulses stimulation tends to decay exponentially with the increasing pulse interval, consistent with the behavior of biological synapses.

The above studies have demonstrated the effective simulation of biological synaptic function and plasticity in ReS_2_/WS_2_‐based planar memristors, all of which are electrical memristive characteristics. Meanwhile, benefiting from the interlayer coupling and charge transfer, ReS_2_/WS_2_ also displays unique photoresponsive behavior as a type II heterostructure,^[^ ^47^ ^]^ based on which the optoelectronics‐inspired memristor characteristics can also be expected. Since the interlayer charge transfer results in a diminution of *V*_set_ in the ReS_2_/WS_2_‐based memristor compared with that of the ReS_2_‐based device (Figure 2d,e), a light modulation is further performed on the two memristors under different excitation wavelengths (532 and 690 nm) and with various optical power densities. The *I–V* curves for all the cases are acquired, as shown in Figures S11 and S12 in the Supporting Information, respectively, and the extracted *V*_set_ values are illustrated in Figure 4g. The results for ReS_2_/WS_2_‐based memristor show interesting wavelength‐dependent conductance controllability. Under a 532 nm excitation, its *V*_set_ drops from about 2.90 to 2.40, 2.00, and 0.90 V for the optical power density of 0, 2.3E‐6, 6.9E‐6, and 1.1E‐5 mW µm^−2^, respectively, exhibiting a negative correlation with the optical power density. While a 690 nm excitation basically does not affect the *V*_set_ that is almost stable at 3.00 V even when the optical power density increases to 1.1E‐5 mW µm^−2^. Different from the wavelength‐dependent performance for heterostructure memristor, the *V*_set_ of ReS_2_‐based device is basically stable at around 3.10 V under illumination with different optical power densities at both wavelengths. The obtained *V*_set_ values are essentially coincident with that measured without the excitation and larger than that of the ReS_2_/WS_2_‐based memristor under the same conditions. This strongly suggests that the variation of *V*_set_ originates from the property of the heterostructure rather than the inherent characteristics of ReS_2_. Compared with the single layer, the formation of type II heterostructure enables optical modulation over its electrical performance. Moreover, the LRS basically can be retained during the optical switching, as shown in Figure S13 in the Supporting Information. Different from most of the existing memristors, which only exhibit electrical tunability,^[^ ^48^, ^49^ ^]^ the above results demonstrate an exciting behavior for the optoelectronic memristor based on the ReS_2_/WS_2_ heterostructure.

The light‐tunable synaptic plasticity is then investigated by applying a pulse under 532 nm excitation at different optical power densities. As shown in Figure 4h, the conductance at all optical power densities maintains an increasing trend with the pulse number and exhibits a considerable light‐sensing behavior. Under the optical power densities of 2.3E‐6, 4.6E‐6, and 6.9E‐6 mW µm^−2^, the maximum/minimum conductance ratio can reach 161, 181, and 200, respectively (the minimum value is 0.0036 µS before the pulse). Such optical power density‐dependent conductance control indicates the optical tunability of the synaptic weight, predicting a potential for future visual neural applications. Figure 4i exhibits an effective control over the required time for the device current to stabilize when a single pulse is applied through the modulation of optical power density. This time should be dependent on the pulse width and interval because continuous or frequent electrical stimulation will vary the response current. For better visibility, a pulse with a reading voltage of 1 V, amplitude of 5 V, and pulse width and interval of both 3 s is applied. It is found that the average required time decreases from 1.8 to 0.7 s with the increase of 532 nm excitation power densities from 0 to 6.9E‐6 mW µm^−2^. This hints at the advanced sensitivity of optically modulated memristors on neuromorphic applications.

**Figure 5:**
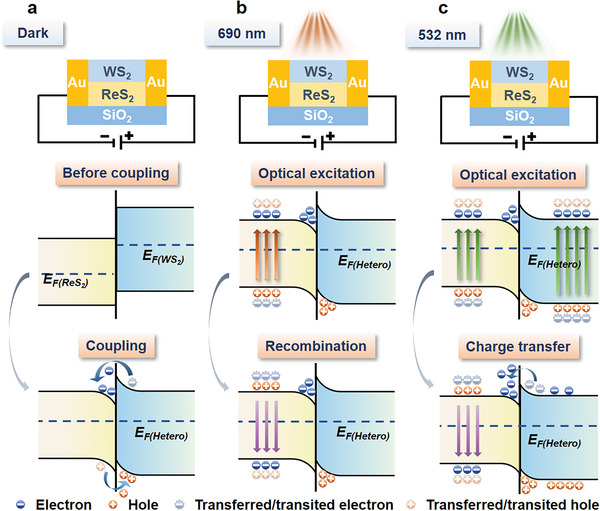
Schematic diagram and band structure evolution of the ReS_2_/WS_2_ heterostructure under the optical modulations with different wavelengths.

One issue that needs to be clarified is the wavelength‐dependent conductance behavior of the ReS_2_/WS_2_‐based planar memristor. From the perspective of band structure, the mechanism can be described in **Figure** **5**. Without the optical modulation, the electrons transfer from WS_2_ to ReS_2_, while the holes transfer in the opposite direction, forming a built‐in electric field directed from WS_2_ to ReS_2_ (Figure 5a). Under a 690 nm (1.80 eV) laser irradiation, monolayer ReS_2_ with a direct bandgap of about 1.65 eV is excited, while WS_2_ with a 2.07 eV bandgap is out of the excitation energy (Figure 5b). Consequently, the produced photogenerated carriers distribute mainly within the ReS_2_ layer. Driven by the built‐in electric field, the electrons tend to transfer to WS_2_, which is blocked by the interfacial potential barrier between the conduction band edges. As a result, the excited electrons and holes will mostly recombine through a radiative or nonradiative process, which essentially does not alter the net carrier distribution in the system. As a result, the *V*_set_ of the ReS_2_/WS_2_‐based memristor is essentially unaffected under the modulation of 690 nm illumination regardless of the optical power. The case of ReS_2_‐based device (Figure 4g, red and blue lines) is similar. For the 532 nm (2.33 eV) laser irradiation, both the ReS_2_ and WS_2_ layers can be excited, as shown in Figure 5c. Since the photon energy is closer to the optical bandgap of WS_2_, a higher excitation efficiency with more photogenerated carriers is produced in WS_2_ than in ReS_2_. The additional electrons will transfer from WS_2_ into ReS_2_ through the heterogenous interface, while the interfacial potential barrier will block the transfer of additional holes. The increased number of electrons in ReS_2_ makes a positive contribution to the resistive switching from HRS to LRS. Consequently, the number of excited electrons increases with increasing optical power density under the 532 nm excitation, and the set voltage (Figure 4g, yellow line) and required time of current stabilization (Figure 4i) are thus reduced.

## 3 Conclusion

In summary,  scalable 2D planar memristors based on ReS_2_/WS_2_ heterostructure are constructed with synaptic plasticity, and their electrical and optical tunability is demonstrated. The type II band structure with strong interlayer interaction is confirmed by Raman, TRPL, HRTEM, and KPFM characterizations for the heterostructure. The fabricated memristors exhibit a unique unipolar resistive switching property, with high *R*_off_/*R*_on_ ratio of up to 10^6^, clearly extended endurance, and long retention time. The set voltage exhibits a decreasing trend with reducing channel length, while the high *R*_off_/*R*_on_ ratio can be well maintained. Based on the clearly defined set and reset processes, the device can successfully simulate partially biological synaptic functions, including STP, SRDP, and PPF, demonstrating the potential applications in neural networks and complex systems. The set voltage can also be effectively tailored in a wide range from 4.50 to 0.40 V by applying a gate voltage, owing to the electrostatic doping or depletion. Besides, optical control over the resistive switching is further achieved by manipulating the interlayer charge transfer within the heterostructure, and the modulation is strongly determined by the excitation wavelength and power. All these findings open up a new gate toward the development of novel devices based on 2D van der Waals heterostructures and push forward the research frontier in neurofunctional devices with optical programmability.

## 4 Experimental Section

### Preparation of ReS_2_/WS_2_ Heterostructure

2D monolayer ReS_2_ and WS_2_ crystals were grown under atmospheric pressure using the CVD growth methods previously reported.^[^ ^37^, ^38^ ^]^ The ReS_2_/WS_2_ heterostructure with a special stacking configuration (60° vertical stacking) was prepared through the alignment transfer with the typical wet transfer method. The poly(methyl methacrylate) (PMMA) was used as the supporting film, assisting in peeling off the monolayer ReS_2_ and WS_2_ from mica and sapphire substrates, respectively.

### Characterizations

The Raman and PL spectra were recorded using a WITec alpha 300RA confocal spectrometer system with a laser wavelength of 488 nm and a beam size of about 1 µm. The step size of the Raman and PL mapping was 800 nm. A SPA400‐Nanonavi AFM was used to measure the thickness of the ReS_2_/WS_2_ heterostructure. TEM measurements, including HRTEM and SAED, were performed on a field‐emission TEM (JEM‐2100) at an accelerating voltage of 200 kV. The surface potential of ReS_2_/WS_2_ was measured by the Kelvin Probe Module of the SPA400‐Nanonavi AFM after it was transferred to an Au‐coated SiO_2_/Si substrate. The photoinduced interfacial charge transfer behavior of the samples was recorded by the TRPL spectra (HORIBA, MicOS) at a PL peak wavelength of 620 nm.

### Device Fabrication and Measurements

ReS_2_/WS_2_, ReS_2_, and WS_2_ layers were transferred to SiO_2_/Si substrates, respectively, through the above transfer method. For ReS_2_/WS_2_ heterostructure, a small region of the top WS_2_ was etched by plasma treatment to expose the bottom ReS_2_. Then the electrodes were defined over the WS_2_ and ReS_2_ areas simultaneously. The ReS_2_/WS_2_‐based devices with channel lengths of 7, 5, and 4 µm, ReS_2_‐based, and WS_2_‐based devices with channel lengths of 7 µm were patterned by the direct‐write laser photolithography technique (MicrowriterML). The ReS_2_/WS_2_‐based devices with a channel length of 500 nm were patterned by the electron beam lithography technique (Sigma300+ELPHY Quantum). After the lithographical processes, 50 nm Au films were deposited by magnetron sputtering to serve as the source and drain electrodes. The fabricated devices were annealed at 200 °C in mixed Ar (a flow rate of 100 sccm) and hydrogen (H_2_) (a flow rate of 3 sccm) gas environment for 30 min to improve the quality of the heterogenous interface as well as the metal‐semiconductor‐contact. The electrical properties of the devices were measured in ambient atmosphere conditions at room temperature under dark and laser irradiation conditions using a semiconductor parameter analyzer (Agilent B2912A source‐meter unit system).

## Conflict of Interest

The authors declare no conflict of interest.

## Supporting information

Supporting Information

## Data Availability

The data that support the findings of this study are available from the corresponding author upon reasonable request.
