# Supplementary material for: Controllable Resistive Switching in ReS2/WS2 Heterostructure for Nonvolatile Memory and Synaptic Simulation
Source: Adv Sci (Weinh). 2023 Aug 2;10(28):2302813. doi: 10.1002/advs.202302813 (PMC10558669; doi:10.1002/advs.202302813)
Supplement: Supplementary file 1 — Supporting Information [file ADVS-10-2302813-s001.pdf]

ADVANCED SCIENCE
Open Access

Supporting Information

for *Adv. Sci.*, DOI 10.1002/advs.202302813

Controllable Resistive Switching in ReS_2/WS_2 Heterostructure for Nonvolatile Memory and Synaptic Simulation

Feihong Huang, Congming Ke, Jinan Li, Li Chen, Jun Yin, Xu Li, Zhiming Wu*, Chunmiao Zhang, Feiya Xu, Yaping Wu* and Junyong Kang**

Supporting Information

Controllable resistive switching in ReS_2/WS_2 heterostructure for non-volatile memory and synaptic simulation

Feihong Huang, Congming Ke, Li Chen, Jun Yin, Xu Li, Zhiming Wu*, Chunmiao Zhang, Feiya Xu, Yaping Wu*, and Junyong Kang**

F. Huang, Dr. C. Ke, J. Li, Dr. X. Li, Prof. Z. Wu, Dr. C. Zhang, F. Xu, Prof. Y. Wu, Prof. J. Kang
Department of Physics, Engineering Research Centre for Micro-Nano Optoelectronic Materials and Devices at Education Ministry, Fujian Provincial Key Laboratory of Semiconductor Materials and Applications, Xiamen University, Xiamen, 361005, P. R. China
E-mail: xuliphys@xmu.edu.cn, zmwu@xmu.edu.cn, ypwu@xmu.edu.cn, jykang@xmu.edu.cn

Dr. L. Chen
Ningbo Institute of Materials Technology and Engineering, Chinese Academy of Sciences, Ningbo, P. R. China

Dr. J. Yin
Pen-Tung Sah Institute of Micro-Nano Science and Technology, Xiamen University, Xiamen 361005, P. R. China

1. Raman and PL intensity mappings and AFM image of the ReS_2/WS_2 heterostructure.

The Raman intensity mappings in **Figure S1**a,b show a uniform contrast throughout the heterostructure, demonstrating the excellent quality of the transferred sample. PL intensity mapping in Figure S1c illustrates a significant fluorescence

quenching owing to the reduced charge recombination in the heterostructure area. Figure S1d displays the AFM image, where the measured heights at the ReS_2 and WS_2 edges are approximately 0.80 nm and 0.85 nm, in proper agreement with their monolayer thickness, respectively.[1] Nevertheless, the step between ReS_2 and ReS_2/WS_2 overlapping regions is slightly smaller than the thickness of monolayer WS_2, suggesting the existence of interlayer interaction. The Raman and PL intensity mapping and AFM image indicate that heterostructure with interlayer interactions is successfully prepared using monolayers ReS_2 and WS_2.

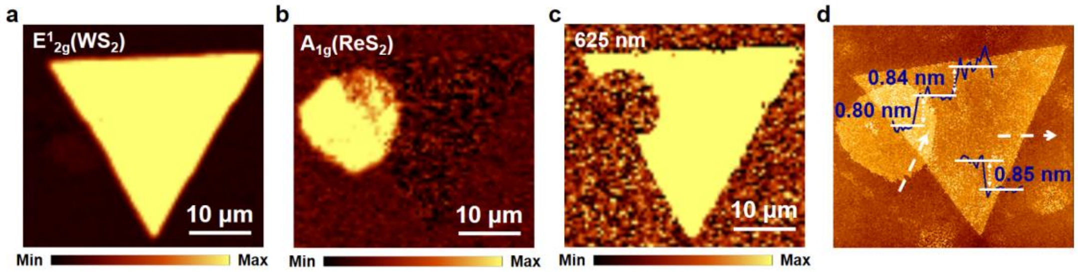

Figure S1. (a, b) Raman intensity mapping at the peak positions of 359 cm^{-1} and 216 cm^{-1}, respectively. (c) PL intensity mapping at the peak position of 625 nm. (d) AFM image of the heterostructure with the height profiles taken along the white dashed arrows.

2. KPFM measurement of the ReS_2/WS_2 heterostructure transferred onto an Au-plated SiO_2/Si substrate.

In the KPFM measurement, the mapping image is obtained through the relation: $\varphi_s = \varphi_{tip} - V_{CPD}$, where φ_{tip} is the work function of the tip, and V_{CPD} is the contact potential difference between the tip and the material.[2] The φ_s is determined only by the Fermi energy levels of the materials, and the Fermi energies of ReS_2 and WS_2 move to relatively equilibrious with Au when they both connect to Au, as shown in **Figure S2**c,d. The φ_s difference between WS_2 and Au is slightly larger than that between ReS_2 and Au, i.e., the potential between WS_2 and Au that needs to be compensated is probably greater. Accordingly, it can be speculated that the Fermi energy of WS_2 is above that of ReS_2. While it has to be mentioned that, since the presence of the Au-plated substrate, charges can transfer between the monolayer

materials and Au. As a result, there are some deviations in their authentic surface potentials. Furthermore, in the heterostructure region, the presence of the interlayer van der Waals barrier may also influence the detected potential difference. Consequently, only the relative values of the φ_s for ReS_2 and WS_2 are concerned.

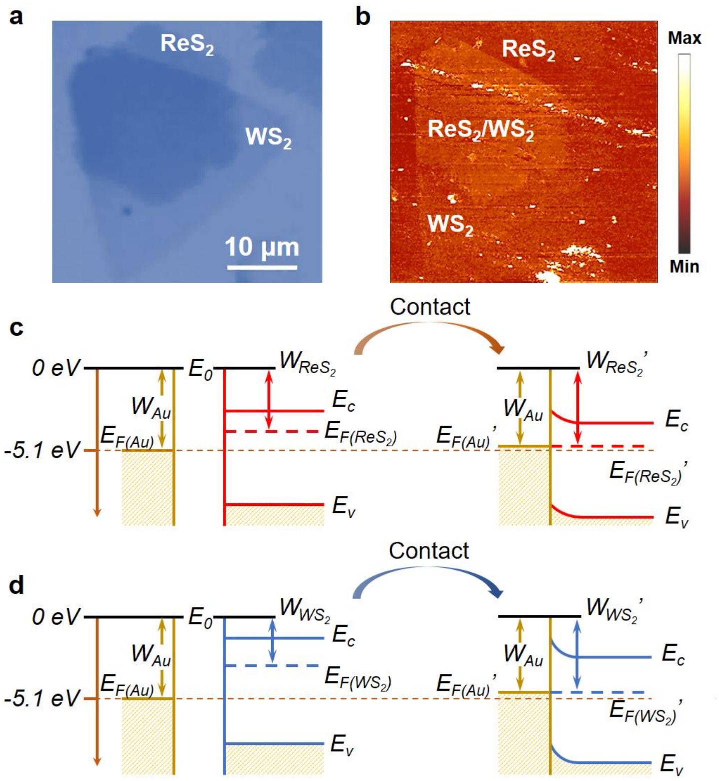

Figure S2. (a) Optical and (b) AFM image of a ReS_2/WS_2 heterostructure transferred to an Au-plated SiO_2/Si substrate for the KPFM measurement. The band alignments of (c) ReS_2/Au and (d) WS_2/Au before and after contact.

3. Lifetime calculations from the TRPL measurements.

To ensure the reliability of the results, both TRPL measurements for WS_2 and ReS_2/WS_2 are performed on two samples for each. **Table S1** provides the fitting parameters of Equation (1) (in the main text) for the decay curves for pristine monolayer WS_2 and the WS_2 in the heterostructure. For pristine monolayer WS_2, the charge transfer item is ignorable. Therefore, only the first two components in Equation (1) are fitted, where the shorter and longer components correspond to the surface state recombination and the radiative recombination, respectively. As for WS_2

in the heterostructure, the extra third component is involved by considering the interlayer charge transfer between WS_2 and ReS_2.[3] The average lifetime in Table S1 is estimated through the relation:[4] $\bar{\tau}=\sum_{i=1}^{n} W_i\tau_i$, where W_i is the weight occupied by the corresponding component τ_i.

Table S1. Lifetimes of the pristine monolayer WS_2 and that in the ReS_2/WS_2 heterostructure.

WS_2	Lifetimes (ps)	Amplitudes	Weight (%)	Average lifetimes (ps)
Sample 1	τ_1=108.825	A_1=0.71218	W_1=82.6904	705.644
	τ_2=3556.75	A_2=0.14908	W_2=17.3095	
Sample 2	τ_1=144.96	A_1=0.83370	W_1=85.9157	695.382
	τ_2=4053.01	A_2=0.13667	W_2=14.0832	
ReS_2/WS_2	Lifetimes (ps)	Amplitudes	Weight (%)	Average lifetimes (ps)
Sample 1	τ_1=99.4973	A_1=0.64911	W_1=62.9501	132.349
	τ_2=99.4975	A_2=0.36591	W_2=35.4856	
	τ_3=2199.63	A_3=0.01613	W_3=1.56427	
Sample 2	τ_1=101.853	A_1=0.68275	W_1=64.0652	135.777
	τ_2=101.853	A_2=0.36749	W_2=34.4831	
	τ_3=2438.83	A_3=0.01547	W_3=1.45161	

4. The first-principles calculations of ReS_2/WS_2 heterostructure.

The band structure of ReS_2/WS_2 heterostructure is simulated based on the density functional theory, as implemented in the Vienna Ab-initio simulation package (VASP) code. A lateral slab model with a 20 Å vacuum layer along the z direction is introduced to avoid the interactions between interlayer and between vacancies. The atomic configuration with the lowest energy is considered only, as shown in Figure S3a. Electron wave functions are expanded in-plane with an energy cutoff of 450 eV. The Brillouin zone is sampled with a $9\times 9\times 1$ Monkhorst-Pack grid of k points. All atomic degrees of freedom, including lattice constants, are fully relaxed with self-consistent convergence criteria of 0.01 eV/Å and 10^{-6} eV for the atomic forces and total energy, respectively. To obtain the property more accurately, the bandgap from the Perdew-Burke-Ernzerhof functional is amended to that of Heyd-Scuseria-Ernzerhof (HSE06).

The band structure of ReS_2/WS_2 heterostructure is shown in Figure S3b. The ReS_2 exhibits a direct bandgap of 1.70 eV (~ 730 nm) at Γ point, and the WS_2 layer shows a direct bandgap of about 1.90 eV (~ 652 nm) is found at K and K' points, well agrees with the PL spectrum in Figure 1c. Moreover, the bottom of the conduction band and top of the valence band are contributed by ReS_2 and WS_2, respectively, exhibiting a type II band alignment and is consistent with the KPFM, TRPL measurements and the proposed contact model in Figure 1.

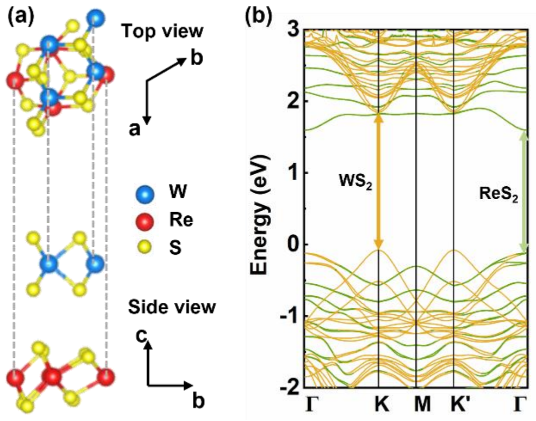

Figure S3. (a) Atomic configuration and (b) the first-principles calculated band structure of ReS_2/WS_2 heterostructure, where the green and yellow bands correspond to ReS_2 and WS_2, respectively.

5. *I-V* characteristics of the ReS_2/WS_2-based memristor.

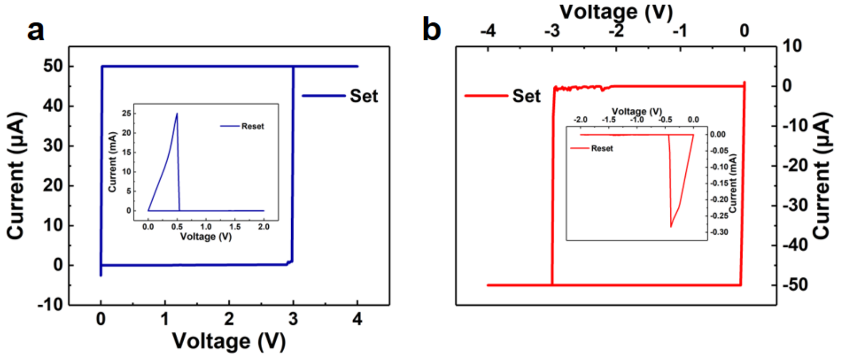

Figure S4. (a, b) Typical unipolar resistive switching *I-V* curves under the positive and negative voltages, respectively, for the ReS_2/WS_2-based planar memristor.

6. Statistical *I-V* measurements of ReS_2/WS_2-based planar memristors with thechannel length of 7 μm.

To obtain the device-to-device statistic results, three ReS_2/WS_2-based memristors with the channel length of 7 μm are fabricated to examine the set voltages (V_{set}) distributions. **Figure S5**a,b shows the unipolar resistive switching *I-V* curve of the other two ReS_2/WS_2-based planar memristors, respectively. Figure S5c,d depict the histogram results of the extracted V_{set} for the devices. A Gaussian fit suggests that the V_{set} generally distributes around 2.87 V and 2.86 V, respectively, which are very close to each other. Moreover, all three ReS_2/WS_2-based memristors exhibit a notable R_{off}/R_{on} ratio higher than 10^6 (Figure S5e). The device-to-device statistic results for V_{set} and R_{off}/R_{on} ratio indicate a good uniformity and stability of the device performance.

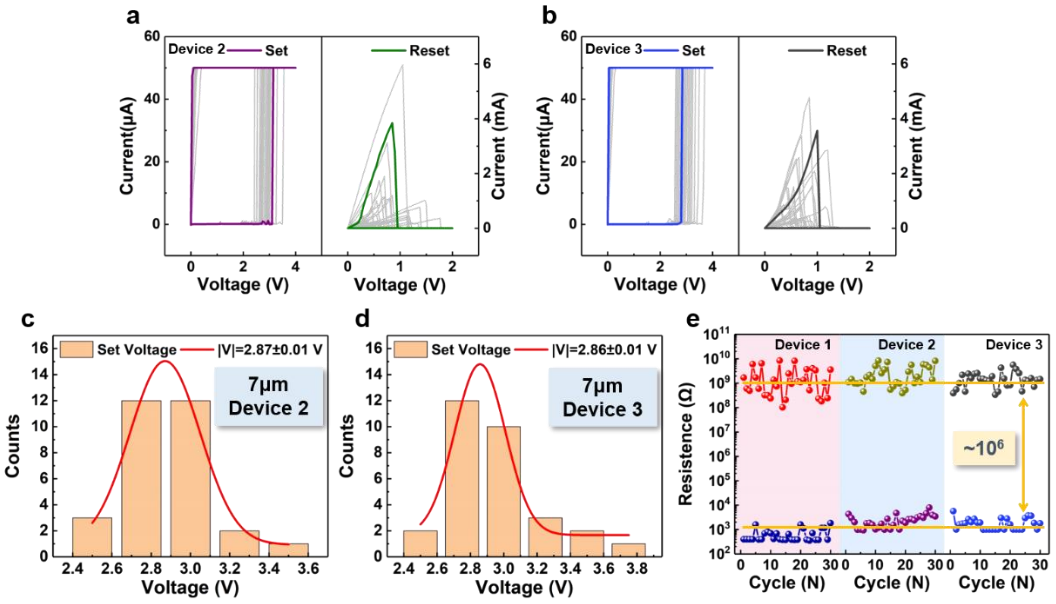

Figure S5. (a, b) Unipolar resistive switching *I-V* curves of other two ReS_2/WS_2-based planar memristors with the channel length of 7 μm, respectively. (The device tested in Figure 2a-d is defined as device 1, and the other two devices tested here are defined as device 2 and 3, respectively) (c, d) Statistical distributions of the V_{set} for devices 2 and 3 based on 30 consecutive switching cycles, respectively. (e) The LRS, HRS, and R_{off}/R_{on} ratio for the three ReS_2/WS_2-based planar memristors from the statistics of 30 consecutive switching cycles.

7. Durable device performance of the ReS_2/WS_2-based memristor.

To investigate the durability of the device, the resistive switching behaviors are examined by comparing the *I-V* properties of the device after placing it in the open air for 1, 47, 69, 195, 281, and 342 days. As shown in **Figure S6**a,b, the good uniformity and stability of the *I-V* curves indicate a durable device performance of the ReS_2/WS_2-based memristor. Moreover, a switching cycling test of the heterostructure memristor is conducted over 200 times (Figure S6c), which shows a reliable resistive switching performance with a clear memory window.

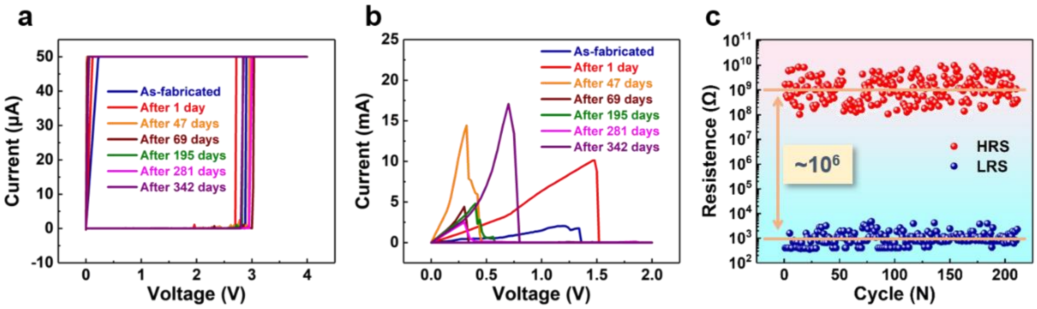

Figure S6. (a, b) *I*-*V* characteristics of the ReS_2/WS_2-based planar memristor exposed in the open air for 1, 47, 69, 195, 281, and 342 days after being fabricated. (c) Statistical analysis of the HRS and LRS over 200 switching cycles for the device.

8. *I*-*V* characteristics of the ReS_2-based and WS_2-based devices.

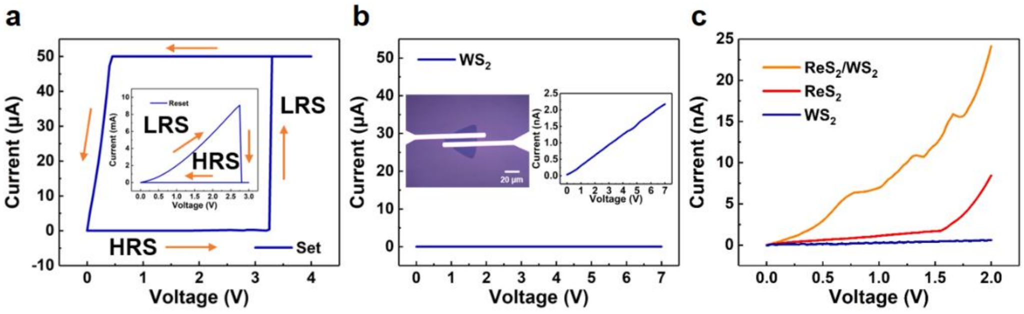

Figure S7. (a) Typical unipolar resistive switching *I*-*V* curve under the positive voltages for the ReS_2-based planar memristor. (b) *I*-*V* curve and the optical image (inset) of the WS_2-based planar device, respectively. (c) *I*-*V* curves of the ReS_2/WS_2, ReS_2, and WS_2 under scanned voltages lower than their setting thresholds.

9. Resistive switching performance of the ReS_2/WS_2-based planar memristor with different channel lengths.

In order to study the influence of device dimension on the performance, ReS_2/WS_2-based memristors with channel lengths of 7 μm, 5 μm, 4 μm, and 500 nm are fabricated. Their resistive switching properties are measured and compared in Figure 2b-d, Figure S8, and Table S2. All the devices exhibit unique unipolar characteristics, and good reliability is also demonstrated during the repeated switching cycles (the gray cycle curves in Figure 2b and Figure S8b,e,h). The V_{set} for all the devices are extracted and depicted in the histogram in Figure 2d and Figure S8c,f,i,

respectively. The V_{set} generally distributes around 2.90 V, 2.76 V, 2.57 V, and 1.21 V, respectively, reduced with the decreasing channel length of the memristor.

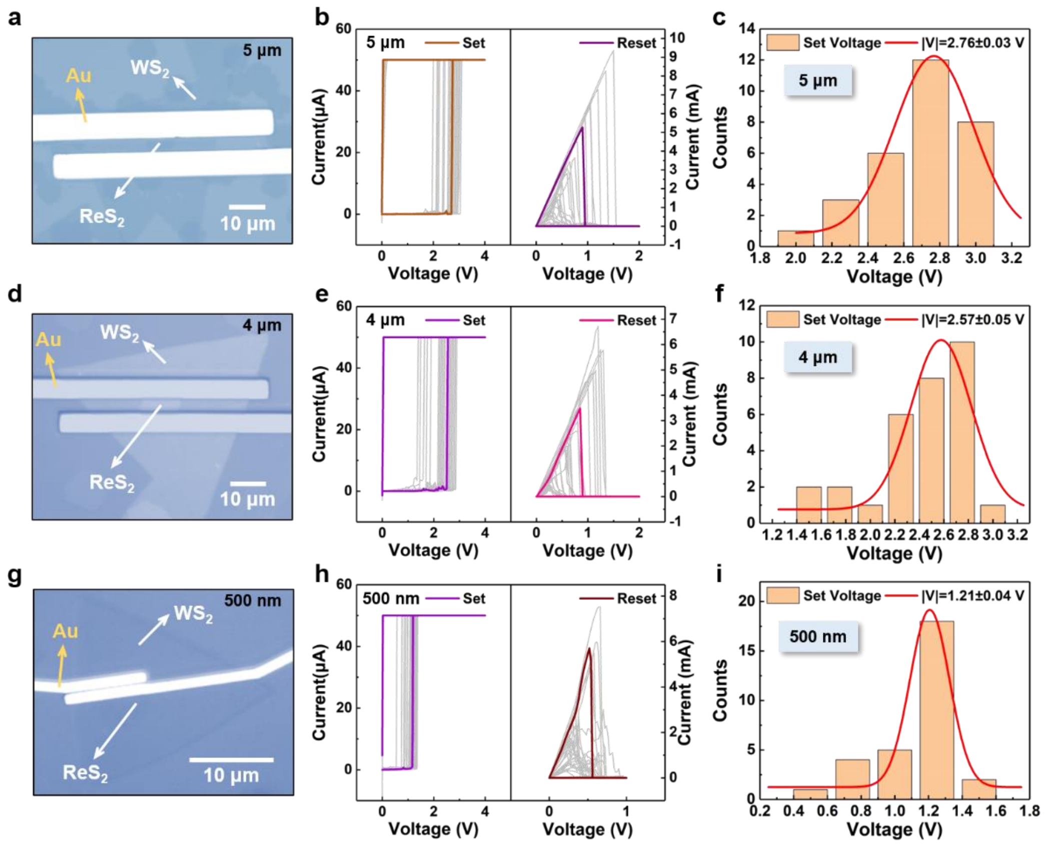

Figure S8. Resistive switching performance of the ReS_2/WS_2-based planar memristors with channel lengths of 5 μm, 4 μm, and 500 nm. (a, d, and g) Optical micrographs, (b, e, and h) *I*-*V* curves, and (c, f, and i) statistical distributions of the V_{set}, respectively.

Table S2. Comparison of the performance of ReS_2/WS_2-based planar memristors with the channel lengths of 7 μm, 5 μm, 4 μm, and 500 nm.

**The ReS_2/WS_2-based two-terminal memristor**				
**Channel lengths**	7 μm	5 μm	4 μm	500 nm
$\mathbf{V_{set}}$	~2.90 V	~2.76 V	~2.57 V	~1.21 V
$\mathbf{R_{off}/R_{on}}$	~10^6	~10^5	~10^5	~10^4

10. Gate modulation on the ReS_2/WS_2-based planar memristor.

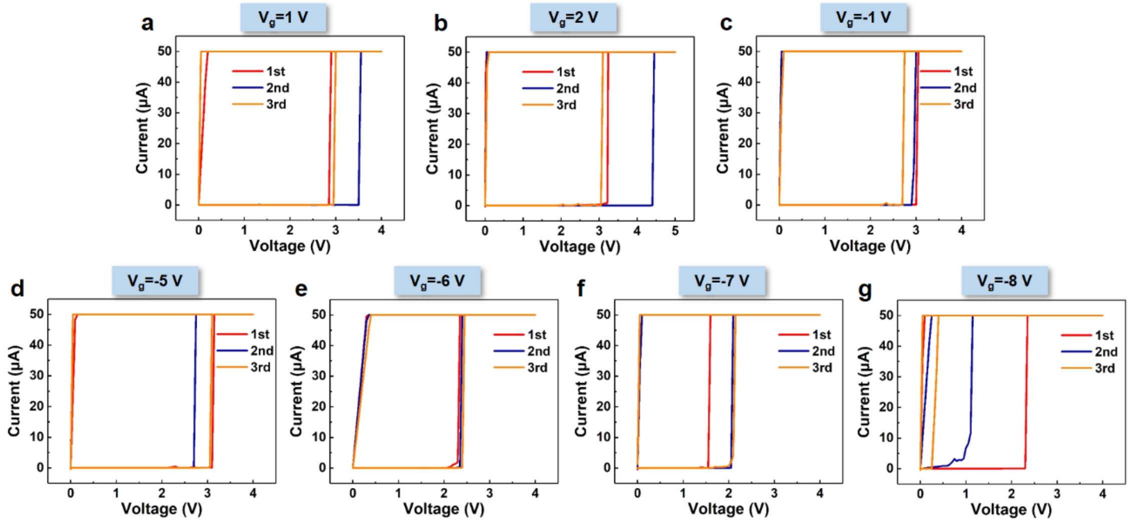

Figure S9. Resistive switching *I-V* curves of the ReS_2/WS_2-based planar memristor under the gate voltages of (a) 1 V, (b) 2 V, (c) -1 V, (d) -5 V, (e) -6 V, (f) -7 V, and (g) -8 V.

11. Conductance modulation on the ReS_2/WS_2-based planar memristor.

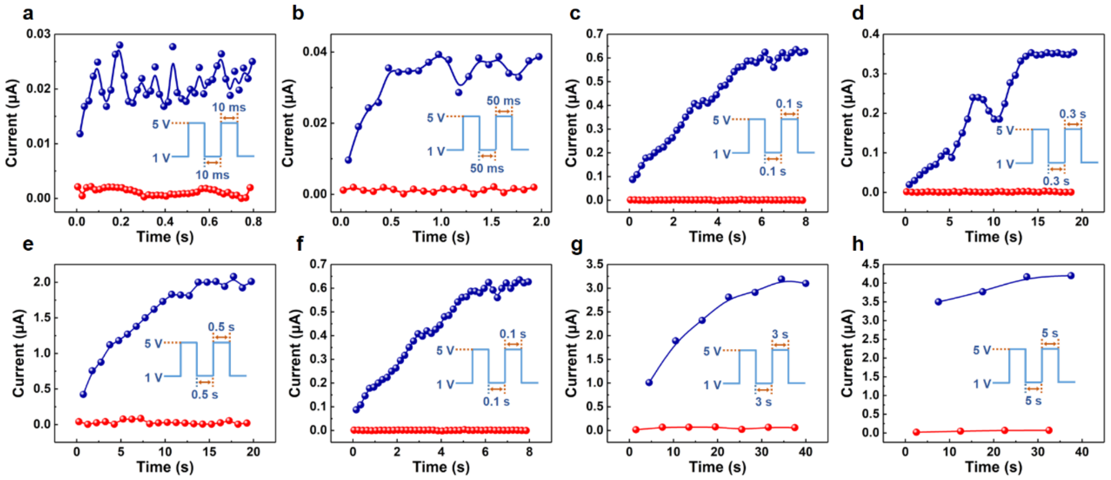

Figure S10. Conductance modulation on the ReS_2/WS_2-based planar memristor with various pulse widths and intervals from 0.01 s to 5 s simultaneously.

12. Optical modulation of the ReS_2/WS_2-based and ReS_2-based planar memristors.

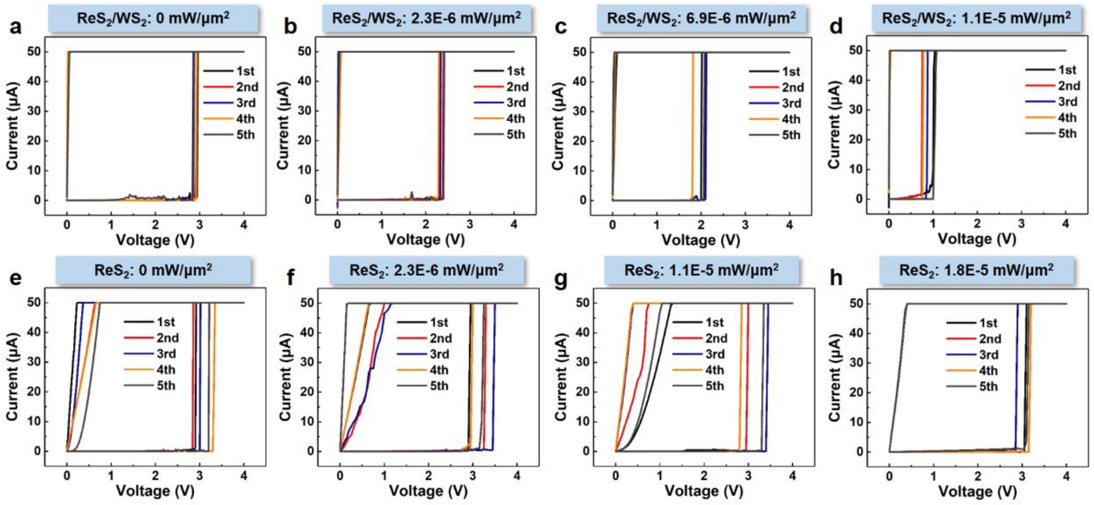

Figure S11. Resistive switching *I-V* curves of the ReS_2/WS_2-based and ReS_2-based planar memristors under a 532 nm laser with different optical power densities.

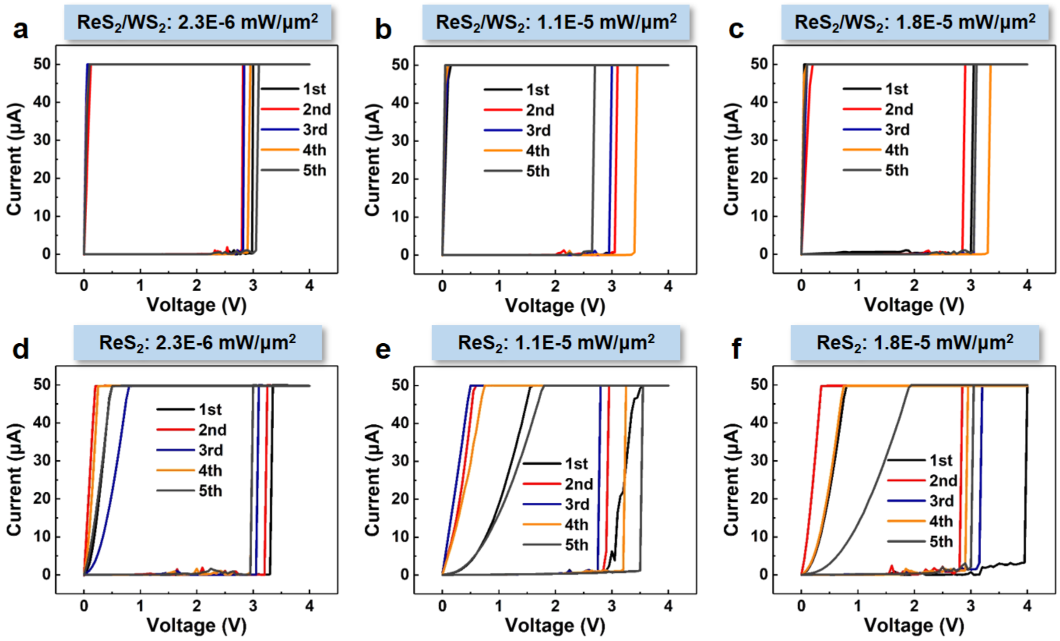

Figure S12. Resistive switching *I-V* curves of the ReS_2/WS_2-based and ReS_2-based planar memristors under a 690 nm laser with different optical power densities.

13. Influence of optical illumination on the resistance state retention of ReS_2/WS_2-based memristor.

The influence of optical illumination on the resistance/conductance retention of the ReS_2/WS_2-based memristor is shown in **Figure S13**. The device is set to an LRS at first. After applying an optical illumination (532 nm, 1.8E-5 mW/μm^2), the LRS remains, while the explicit value of the conductance shows a little increase. As further removing the optical illumination, the LRS is also unchanged except for a slight conductance decrease to near the initial value. Therefore, the resistance state can basically be retained during the optical switching. But meanwhile, the optical illumination could also modulate the exact conductance in an appropriate range, which provides the possibility for tuning the threshold voltage of the memristor and its synaptic plasticity when applied in the neural networks.

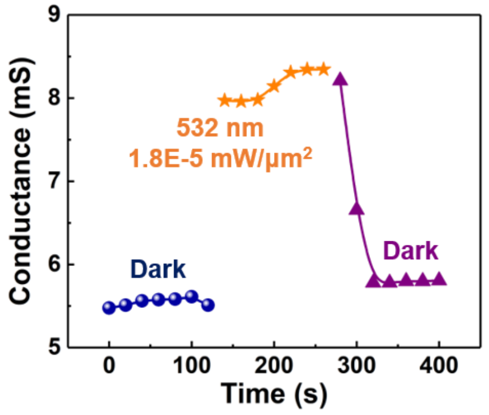

Figure S13. The conductance of the ReS_2/WS_2-based memristor in an LRS without and with optical illumination.

References

[1] F. Cui, C. Wang, X. Li, G. Wang, K. Liu, Z. Yang, Q. Feng, X. Liang, Z. Zhang, S. Liu, Z. Lei, Z. Liu, H. Xu, J. Zhang, *Adv. Mater.* **2016**, *28*, 5019.

[2] I. Sharma, B. R. Mehta, *J. Alloys Compd.* **2017**, *723*, 50.

[3] Y. Yuan, X. Zhang, H. Liu, T. Yang, W. Zheng, B. Zheng, F. Jiang, L. Li, D. Li, X. Zhu, A. Pan, *J. Alloys Compd.* **2020**, *815*, 152309.

[4] Q. Zhu, Z. Wang, X. Cai, W. Wang, G. Wu, L. Kong, X. Zheng, Y. Cao, Y. Wu, X. Li, Z. Wu, J. Kang, *J. Power Sources* **2020**, *465*, 228251.
